# Increasing the High Throughput of a Luminescence-Based Serum Bactericidal Assay (L-SBA)

**DOI:** 10.3390/biotech10030019

**Published:** 2021-09-18

**Authors:** Maria Grazia Aruta, Martina Carducci, Francesca Micoli, Francesca Necchi, Omar Rossi

**Affiliations:** GSK Vaccines Institute for Global Health (GVGH) S.r.l., 53100 Siena, Italy; maria-grazia.x.aruta@gsk.com (M.G.A.); martina.x.carducci@gsk.com (M.C.); francesca.x.micoli@gsk.com (F.M.); francesca.x.necchi@gsk.com (F.N.)

**Keywords:** serum bactericidal assay, vaccine, functional assay, high throughput, luminescent SBA

## Abstract

Serum bactericidal assay (SBA) is the method to investigate in vitro complement-mediated bactericidal activity of sera raised upon vaccination. The assay is based on incubating the target bacteria and exogenous complement with sera at different dilutions and the result of the assay is represented by the sera dilution being able to kill 50% of bacteria present in the inoculum. The traditional readout of the assay is based on measurement of colony-forming units (CFU) obtained after plating different reaction mixes on agar. This readout is at low throughput and time consuming, even when automated counting is used. We previously described a novel assay with a luminescence readout (L-SBA) based on measurement of ATP released by live bacteria, which allowed to substantially increase the throughput as well as to reduce the time necessary to perform the assay when compared to traditional methods. Here we present a further improvement of the assay by moving from a 96-well to a 384-well format, which allowed us to further increase the throughput and substantially reduce costs while maintaining the high performance of the previously described L-SBA method. The method has been successfully applied to a variety of different pathogens.

## 1. Introduction

Serum bactericidal assay (SBA) represents a method to determine in vitro the ability of antibodies present in serum to kill bacteria through complement activation. The assay has been established as an in vitro correlate of protection for bacterial vaccines against cholera [1] and meningococcal disease [2], and is widely used to evaluate functionality of sera raised against pathogens for which a functional assay has not been yet defined as a correlate of protection [3].

In the SBA, bacteria are mixed with dilutions of heat-inactivated serum in the presence of exogenous complement. The number of live bacteria is determined at each serum dilution after a certain amount of time. The dilution of serum resulting in killing 50% of bacteria in the reaction represents the bactericidal antibody titer [4,5].

The traditional SBA methods had some bottlenecks, mainly represented by the need for manually plating onto agar plates and counting the colony-forming units (CFU) both at the beginning and at the end of incubation at each serial dilution. Thus, the assay is considered time consuming and labor intensive for screening large datasets, even when automated colony-counting systems are in place.

In order to overcome these issues, several groups have worked in increasing throughput [6,7]. We developed a luminescence-based high-throughput SBA (L-SBA) in 96-well format. Indeed, in our assay, the reaction mix is directly mixed with BacTiter-Glo Reagent (Promega, Madison, WI, USA), containing a thermostable luciferase and its substrate luciferin that is oxidized and thus emits light in the presence of bacterial ATP. Thus, the level of metabolic ATP released by bacteria surviving the complement-mediated killing can be detected by measuring the level of luminescent signal, which is directly proportional to the number of living bacteria in the assay wells and inversely proportional to the level of functional antibodies that are present in the serum. Hence, in the L-SBA setup, the bactericidal titer can be calculated directly at the end of the bactericidal reaction by reading the microplate in a luminometer, without the need to plate and count CFU. We demonstrated the performance of this method and the equivalence of results compared to the traditional CFU-based method against several pathogens, including *Citrobacter freundii*, *Salmonella* serovars Typhimurium and Enteritidis, *Shigella flexneri* serotypes 2a and 3a, *Shigella sonnei*, *Neisseria meningitidis* [8] and *S.* Paratyphi A [9]. We have also characterized the assay in an intralaboratory manner in terms of specificity, linearity and precision by using human sera raised against an *S. sonnei* GMMA-based vaccine (1790GAHB) as model, demonstrating high performance of L-SBA and further optimizing the analysis method [10]. The L-SBA method has already been extensively applied to evaluate functionality of both preclinical [11,12,13] and clinical sera from our [14] and other groups’ studies [15].

Here, we present a further improvement of the L-SBA method in terms of throughput by moving from a 96-well to a 384-well format. We demonstrated consistent results and high correlation between serum titers obtained using the two L-SBA formats against different bacteria: *S. sonnei*, *S. flexneri* 1b, *S. flexneri* 2a, *S. flexneri* 3a, *S.* Typhimurium, *S.* Enteritidis, *S.* Paratyphi A and *C. freundii*. All of those pathogens represent the etiological agent of large, and often underestimated, disease burdens in low- and middle-income countries, especially in children under the age of five. *Shigella* and *Salmonella* cause significant diarrheal disease resulting in illness and death mostly in low-income countries [16]. Shigellosis is the second-leading cause of diarrheal-related mortality, with >200,000 deaths per year, globally [17]; invasive non-typhoidal *Salmonella* (iNTS) disease is a leading cause of morbidity and mortality among infants and HIV-positive adults in sub-Saharan Africa, with an up to 30% mortality rate [18]; enteric fever caused by *S. enterica* serovar Typhi and Paratyphi A is a bacteremic disease with clinical features different from those of other Gram-negative bacteremias [19]. Typhoid fever is most prevalent among children living in areas of Asia and Africa especially, where access to clean water and adequate sanitation is limited, but it is also an important travel-associated disease [20]. Based on clinical severity, disease burden and emergence of antimicrobial resistance, *Shigella* and *Salmonella* are prime targets for vaccine development [21,22,23]. The improvement of SBA in terms of throughput results is considered to be very important for the development of vaccines against both *Shigella* and *Salmonella* enteric diseases pathogens [24,25,26].

## 2. Materials and Methods

### 2.1. Bacterial Strains and Reagents

Bacterial strains used in this work are listed in Table 1. They were stored in glycerol stocks at −80 °C until use.

An overnight culture (16 h at 37 °C, shaking at 180 rpm) was started from a loop of material from frozen stocks in Luria Bertani (LB) medium (Sigma-Aldrich, Saint Louis, MO, USA), supplemented with 20 µg/mL of chloramphenicol (Sigma-Aldrich, Saint Louis, MO, USA) only in the case of the *S. sonnei* strain. The bacterial suspension was then diluted in fresh LB to start a bacterial culture from an optical density (OD_600_) of 0.05 at 600 nm and incubated at 37 °C with 180 rpm agitation, until it reached 0.20–0.25 OD_600_.

### 2.2. Serum Samples

The serum samples used were polyclonal sera raised in mice or rabbits immunized with glycoconjugates (Vi-CRM197 [32,33] and O:2-CRM197 [34]), or with GMMA-based vaccines obtained from *S. flexneri* 1b, 2a, 3a, *S. sonnei*, *S*. Typhimurium and *S*. Enteritidis GMMA-producing strains [11,24,25,26,31,35].

All sera tested in L-SBA were heat-inactivated (HI) at 56 °C for 30 min to remove endogenous complement activity prior to performing the L-SBA.

### 2.3. Luminescent-Based SBA (L-SBA) in 96- and 384-Well Plates

L-SBA was conducted in a 96-well plate (100 μL volume reaction mix containing 25,000 bacteria) format under the same assay conditions and reagent proportions as previously described [8,9] with an optimized method for raw data fitting [10]. Baby rabbit complement (BRC) from Cedarlane (Euroclone) was used as an exogenous complement source (20% BRC in the case of *S. sonnei*, 15% in the case of *S. flexneri* 1b and 3a, 7.5% in the case of *S. flexneri* 2a, 50% in the case of *S.* Typhimurium and Enteritidis, 20% in the case of *S.* Paratyphi A and 5% in the case of *C. freundii*). Phosphate-buffered saline (PBS) and LB medium (only in the case of *S. flexneri* 1b strain) were used for serum and bacteria dilutions for preparation of the reaction mix.

Initially, L-SBA using 384-well plates was performed in the same experimental conditions established for 96-well plates. After the initial bridging, the L-SBA in 384-well format was performed using the same proportion of reagents as for 96-well plates, but in 50 μL final volume.

Up to eight independent replicates of heat-inactivated test sera were serially diluted 11 times, 3-fold apart in 96-well Corning plate.

An additional well containing buffer only was also added as negative control and was used for fitting purposes [10]. Furthermore, ratio of luminescence detected at T180 in wells containing buffer and BRC only and luminescence at T0 is used to evaluate the optimal growth in the assay and as quality control to validate the assay [10].

Heat-inactivated sera, exogenous BRC and diluted bacteria were mixed and incubated for 180 min at 37 °C. At the end of the incubation, the plate containing the assay reaction was centrifuged at 25 °C (room temperature, RT) for 10 min at 4000× *g*. The supernatant was discarded to remove bacterial debris, dead bacteria and the other SBA reagents (for this step, direct aspiration using an automated liquid handler or a plate washer was implemented); the bacterial pellet was resuspended in PBS, transferred to white round-bottom 96- or 384-well plates (Greiner Bio-One, Kremsmünster, Austria) and mixed 1:1 (*v*:*v*) with BacTiter-Glo Reagent (Promega, Madison, WI, USA). After 5 min of incubation at RT on an orbital shaker, the luminescence signal was measured by a luminometer (Synergy Biotek, Winooski, VT, USA).

### 2.4. Calculations

For data analysis, a 4-parameter non-linear regression was applied to raw luminescence data obtained at different dilutions tested for each serum sample.

Fitting was performed by weighting the data for the inverse of luminescence^2, as previously described [10].

GraphPad Prism (GraphPad Software, La Jolla, CA, USA) was used for fitting and IC50 determination. IC50 corresponds to the reciprocal serum dilution necessary to obtain 50% bacterial growth inhibition (SBA titer).

## 3. Results

### 3.1. Moving from 96- to 384-Well Plates

After having verified the feasibility of performing L-SBA in 384-well plates by using the same experimental conditions (bacteria dilution, final volume reaction, BRC percentage, sera dilution volume and method for dispensing buffers or removing supernatants) established with 96-well-plates L-SBA (data not shown), we optimized the 384-well format using half of the reaction volume used for the 96-well format (50 µL rather than 100 µL, maintaining same proportion of reagents).

### 3.2. Comparison between L-SBA in 96- and 384-Well Plates

The relative performance of 384-well-plate L-SBA was evaluated by comparing results obtained with this method to the results of 96-well-plate L-SBA for sera raised against multiple bacteria, such as *S. flexneri* serotypes 1b, 2a and 3a, *S. sonnei* and *S.* serovars Typhimurium, Enteritidis and Paratyphi A.

**Figure 1:**
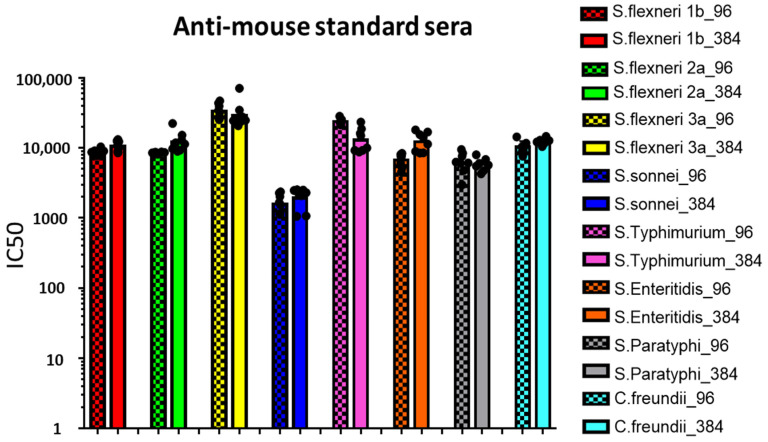
L-SBA titers (IC50) against *S. flexneri* serotypes 1b, 2a and 3a, *S. sonnei*, *S.* serovars Typhimurium, Enteritidis and Paratyphi A and *C. freundii* strains measured in mouse reference sera. Dots represent IC50 values corresponding to each replicate, while bars represent the related geometric means. Checkered bars represent data deriving from 96-wells-plate L-SBA, while solid bars represent data deriving from 384-wells-plate L-SBA.

**Figure 2:**
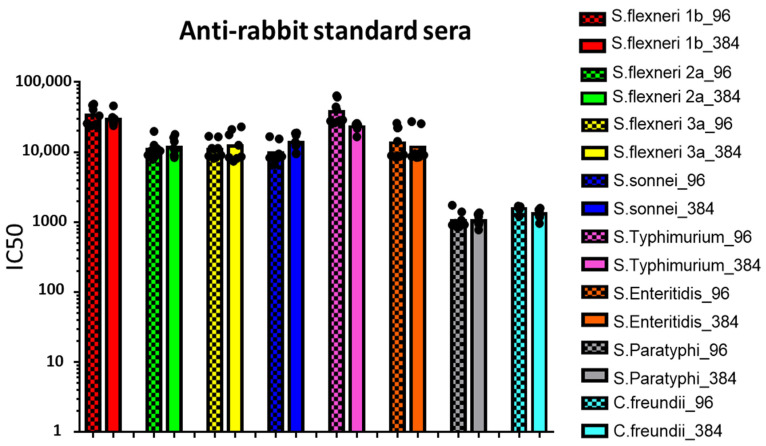
L-SBA titers (IC50) against *S. flexneri* serotypes 1b, 2a and 3a, *S. sonnei*, *S.* serovars Typhimurium, Enteritidis and Paratyphi A and *C. freundii* strains measured in rabbit reference sera. Dots represent IC50 values corresponding to each replicate while bars represent the related geometric means. Checkered bars represent data deriving from 96-wells-plate L-SBA, while solid bars represent data deriving from 384-wells-plate L-SBA.

Mouse (Figure 1) and rabbit (Figure 2) reference sera were tested against the homologous strains in seven or eight independent replicates with each bactericidal reaction simultaneously assayed by both 96-well and 384-well L-SBA.

Similar SBA titers (IC50) were obtained by testing the same reference sera multiple times with both 96- and 384-well L-SBA format (Figure 1 and Figure 2). Moreover, very low variability in the measured IC50 was observed between the two different L-SBA formats, with standard error (SE) among replicates being less than 20% and in the majority of test samples around 10% (Table 2).

**Figure 3:**
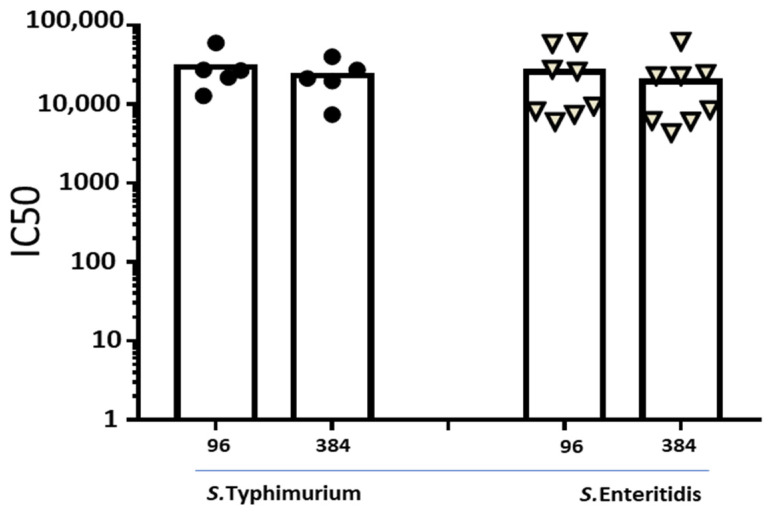
L-SBA titers (IC50) against *S.* Typhimurium and *S.* Enteritidis strains calculated on single sera obtained after immunization of New Zealand White rabbits with a mix of individually formulated *S.* Typhimurium and *S.* Enteritidis GMMA vaccine obtained on day 14, 28, 42 and 71. Dots represent serum samples from individual rabbits.

Finally, to demonstrate equivalence of results obtained by 96- and 384-wells-plate L-SBA in the presence of the intrinsic biological variability of animal response against the same immunogen, individual rabbit sera raised against *S.* Typhimurium or *S.* Enteritidis GMMA (Figure 3) were directly compared using the two methods. By applying paired non-parametric Wilcoxon test, we did not show statistical difference between the two L-SBA methods (*p* = 0.1875 and *p* = 0.1094 for *S.* Typhimurium and *S.* Enteritidis, respectively).

## 4. Discussion

Serum bactericidal assay (SBA) is the method of choice to investigate in vitro complement-mediated bactericidal activity of antibodies present in sera, especially induced upon immunization [4,36,37]. The traditional SBA method is CFU-based, and thus depends on the laborious practice of plating bacteria on solid media at the end of the assay reaction, requiring an overnight incubation and afterwards CFU counting, so it is time consuming and at low throughput.

To overcome those bottlenecks, several groups have worked in increasing throughput by developing both conventional CFU-based assays implementing automated CFU counting [6] or non-conventional SBA by measuring cellular respiration as a survival readout [38]. We have developed a luminescence-based high-throughput SBA method based on luminescence readout (L-SBA) and direct measurement of ATP released by live bacteria on the 96-well format. This method is highly reproducible and has a strong correlation between SBA titers (IC50) determined with traditional CFU counting method [8]. L-SBA has been applied to determine the functionality of sera raised against a broad range of bacterial targets, both at preclinical [11,12,13] and clinical levels [14,15]. The sensitivity of the L-SBA has been evaluated as part of an in depth characterization of the assay (data not shown) performed for each serotype: L-SBA was able to efficiently, specifically and sensibly discriminate between positive and negative samples under the same assay conditions used here.

In this work, we have shown a further optimization of the L-SBA method by adapting the 96-well L-SBA to the 384-wells-plate format.

We demonstrated a good concordance of results obtained using 96-well and 384-well L-SBA formats in all the cases analyzed: (1) against multiple clinically relevant enteric bacterial strains (*S. sonnei*, *S. flexneri* 1b, *S. flexneri* 2a, *S. flexneri* 3a, *S.* Typhimurium, *S.* Enteritidis, *S.* Paratyphi A and C. *freundii)*; (2) using sera raised in two animal species (mice and rabbits); (3) testing independent replicates of the same reference sera or directly comparing the functionality of multiple individual single sera raised against the same vaccine.

A direct comparison of the three methods (traditional CFU-based with manual counting, 96-wells-plate luminescence-based and 384-wells-plate luminescence-based SBA) in terms of performances is shown in Table 3.

In the case of 384-well L-SBA, the assay time remained the same as the 96-well L-SBA with an apparent increase of individual plate reading. However, it needs to be considered that a 384-well plate can accommodate four 96-well-plate layouts; therefore, with the new format, the time for reading a single layout remained basically equivalent.

The main achievements of 384-well L-SBA format are represented by the increase of throughput per day per operator, which goes from 88 to 188 individual sera. This increase is paralleled by a relevant cost reduction (decreasing from about twelve to around eight EUR for each serum assayed) due mostly to the reduction of the reaction volume. During the optimization, we also gained an increase of operator independence, due to the implementation of the automatic liquid handler/plate washer to discard reaction mix prior to the reading, applicable both to 96- and 384-wells L-SBA format.

Overall, the assay costs of reagents were higher for L-SBA compared to traditional SBA; however, the increased throughput of L-SBA method allows one to substantially reduce the labor costs, making the use of the L-SBA method, especially in the 384-well format, attractive and competitive in terms of costs, overall very similar to the ones for the traditional method.

Thus, 384-well-plate L-SBA represents a promising assay particularly for very large-scale studies, as this allows significant savings in terms of costs, time and human resources while maintaining the high performances of the previously developed and well-established 96-well-format L-SBA method. This increase in throughput is particularly important to analyze sera from clinical trials, and it opens the opportunity to analyze a larger number of sera, also against more than one strain, as the assay requires less sera volume. Therefore, this assay will be critical to support the development of vaccines against multiple bacterial targets.

## Figures and Tables

**Table 1:** Bacterial strains used in this study.

Species and Serovar	Strain	Characteristics	Reference(s)
*Shigella flexneri* serotype 1b	140	Clinical isolate	Public Health England (PHE)
*Shigella flexneri* serotype 2a	142	Clinical isolate	Public Health England (PHE)
*Shigella flexneri* serotype 3a	144	Clinical isolate	Public Health England (PHE)
*Shigella sonnei*	71	*S. sonnei* 53G ΔvirG::cat	[26]
*Citrobacter freundii*	NVGH328	Clinical isolate from Novartis Master Culture	[27]
*Salmonella* enterica serovar Typhimurium	D23580	Clinical isolate from blood culture, Malawi	[28,29]
*Salmonella* enterica serovar Enteritidis	CMCC4314	(corresponding to ATCC4931) obtained from the Novartis Master Culture Collection (NMCC)	[30]
*Salmonella* Paratyphi A	NVGH308	Invasive isolates, Nepal	[31]

**Table 2:** L-SBA titers (IC50) against homologous bacteria tested as determined by L-SBA in 96- and 384-well plates in multiple independent replicates (Rep.).

			L-SBA Titers (IC50)				L-SBA Titers (IC50)	
			96-Wells-Plate SBA	384-Wells-Plate SBA			96-Wells-Plate SBA	384-Wells-Plate SBA
***S. flexneri*** **1b Strain**	**Mouse Standard Antiserum**	**Rep. 1**	8126	8490	**Rabbit Standard Antiserum**	**Rep. 1**	24,732	29,776
		**Rep. 2**	7738	12,255		**Rep. 2**	25,699	31,448
		**Rep. 3**	8257	10,670		**Rep. 3**	33,031	26,493
		**Rep. 4**	9092	8694		**Rep. 4**	46,997	28,192
		**Rep. 5**	8697	9869		**Rep. 5**	48,571	24,008
		**Rep. 6**	10,322	8784		**Rep. 6**	40,635	25,847
		**Rep. 7**	8965	13,089		**Rep. 7**	23,901	45,903
		**Rep. 8**	8533	12,554		**Rep. 8**		28,454
		**GeoMean**	8687	10,406		**GeoMean**	33,418	29,456
		**SE**	260	621		**SE**	3481	2258
		**SE%**	3	6		**SE%**	10	8
***S. flexneri*** **2a strain**	**Mouse standard antiserum**	**Rep. 1**	8119	8905	**Rabbit Standard antiserum**	**Rep. 1**	8770	8380
		**Rep. 2**	8473	9682		**Rep. 2**	11,246	9054
		**Rep. 3**	8477	22,253		**Rep. 3**	12,632	18,019
		**Rep. 4**	8450	15,172		**Rep. 4**	19,843	14,242
		**Rep. 5**	8657	9233		**Rep. 5**	10,420	8821
		**Rep. 6**	8741	11,761		**Rep. 6**	10,428	16,238
		**Rep. 7**	8591	11,323		**Rep. 7**	9104	10,757
		**Rep. 8**	8376	12,974		**Rep. 8**	7926	11,323
		**GeoMean**	8484	12,116		**GeoMean**	10,863	11,652
		**SE**	63	1456		**SE**	1243	1206
		**SE%**	1	12		**SE%**	11	10
***S. flexneri*** **3a Strain**	**mouse Standard Antiserum**	**Rep. 1**	46,819	24,862	**Rabbit standard antiserum**	**Rep. 1**	8811	12,624
		**Rep. 2**	25,205	70,660		**Rep. 2**	11,374	21,178
		**Rep. 3**	43,233	56,883		**Rep. 3**	11,343	23,076
		**Rep. 4**	27,388	40,699		**Rep. 4**	16,600	17,792
		**Rep. 5**	39,309	25,032		**Rep. 5**	16,928	7450
		**Rep. 6**	34,009	24,001		**Rep. 6**	9086	8619
		**Rep. 7**	32,125	24,675		**Rep. 7**	8417	8149
		**Rep. 8**	26,146	23,684		**Rep. 8**	8141	8387
		**GeoMean**	33,454	33,063		**GeoMean**	10,897	12,171
		**SE**	2691	6030		**SE**	1180	2110
		**SE%**	8	18		**SE%**	11	17
***S. sonnei*** **Strain**	**Mouse Standard Antiserum**	**Rep. 1**	1658	2517	**Rabbit standard antiserum**	**Rep. 1**	6630	17,512
		**Rep. 2**	1150	2290		**Rep. 2**	8097	9570
		**Rep. 3**	2357	2081		**Rep. 3**	8673	12,372
		**Rep. 4**	1529	2460		**Rep. 4**	9433	18,587
		**Rep. 5**	1139	2489		**Rep. 5**	16,669	18,851
		**Rep. 6**	1121	1068		**Rep. 6**	15,471	9528
		**Rep. 7**	2014	2160		**Rep. 7**	8789	12,956
		**Rep. 8**	2258	1054		**Rep. 8**	8342	14,005
		**GeoMean**	1585	1912		**GeoMean**	9770	13,721
		**SE**	168	201		**SE**	1219	1250
		**SE%**	11	11		**SE%**	12	9
***S.*** **Typhimurium strain**	**Mouse standard antiserum**	**Rep. 1**	28,133	16,038	**Anti-rabbit standard serum**	**Rep. 1**	61,185	25,018
		**Rep. 2**	21,515	23,282		**Rep. 2**	40,788	24,404
		**Rep. 3**	21,259	9986		**Rep. 3**	27,883	25,895
		**Rep. 4**	22,669	14,926		**Rep. 4**	28,813	23,882
		**Rep. 5**	25,324	18,654		**Rep. 5**	44,251	23,305
		**Rep. 6**	23,509	9197		**Rep. 6**	27,725	21,824
		**Rep. 7**	23,182	9208		**Rep. 7**	26,312	16,567
		**Rep. 8**		8749		**Rep. 8**	63,920	23,675
		**GeoMean**	23,557	12,905		**GeoMean**	37,765	22,888
		**SE**	783	1777		**SE**	5077	956
		**SE%**	3	14		**SE%**	13	4
***S.*** **Enteritidis strain**	**Mouse standard antiserum**	**Rep. 1**	7984	18,043	**Rabbit standard antiserum**	**Rep. 1**	14,317	8527
		**Rep. 2**	8327	11,270		**Rep. 2**	8448	9505
		**Rep. 3**	6037	16,781		**Rep. 3**	8931	8681
		**Rep. 4**	7377	13,950		**Rep. 4**	22,548	27,381
		**Rep. 5**	5105	15,416		**Rep. 5**	25,966	25,482
		**Rep. 6**	4482	8436		**Rep. 6**	22,255	9927
		**Rep. 7**	7463	8867		**Rep. 7**	9179	9091
		**Rep. 8**	7867	8457		**Rep. 8**	8701	8442
		**GeoMean**	6683	12,116		**GeoMean**	13,532	11,793
		**SE**	475	1293		**SE**	2448	2675
		**SE%**	7	11		**SE%**	18	23
***S.*** **Paratyphi strain**	**Mouse standard antiserum**	**Rep. 1**	6012	6008	**Rabbit standard antiserum**	**Rep. 1**	1400	941
		**Rep. 2**	2962	4244		**Rep. 2**	851	1296
		**Rep. 3**	6090	6353		**Rep. 3**	865	974
		**Rep. 4**	7579	6832		**Rep. 4**	968	998
		**Rep. 5**	9471	5474		**Rep. 5**	1748	1361
		**Rep. 6**	8381	5657		**Rep. 6**	1065	775
		**Rep. 7**	4740	4694		**Rep. 7**	924	1201
		**Rep. 8**	6259	7945		**Rep. 8**	912	1026
		**GeoMean**	6107	5798		**GeoMean**	1058	1056
		**SE**	680	389		**SE**	105	65
		**SE%**	11	7		**SE%**	10	6
***C. freundii*** **strain**	**Mouse standard antiserum**	**Rep. 1**	11,600	12,664	**Rabbit standard antiserum**	**Rep. 1**	1216	959
		**Rep. 2**	9214	10,450		**Rep. 2**	1579	1317
		**Rep. 3**	14,273	11,422		**Rep. 3**	1693	1503
		**Rep. 4**	9719	12,792		**Rep. 4**	1665	1490
		**Rep. 5**	10,345	14,563		**Rep. 5**	1634	1588
		**Rep. 6**	8914	12,121		**Rep. 6**	1629	1222
		**Rep. 7**	7585	11,946		**Rep. 7**	1539	1300
		**Rep. 8**		12,104		**Rep. 8**		1195
		**GeoMean**	10,052	12,209		**GeoMean**	1557	1307
		**SE**	710	393		**SE**	53	67
		**SE%**	7	3		**SE%**	3	5

**Table 3:** Estimation of throughput comparing traditional CFU-based method with 96- and 384-well L-SBA.

	Traditional CFU-Based SBA	96-Wells-Plate L-SBA	384-Wells-Plate L-SBA
**Final Volume Reaction**	100 µL/well	100 µL/well	50 µL/well
**Assay Time**	1.5 working day	6 h	6 h
**Plate Reading**	2–3 h/SBA plate	2 min/SBA plate	5 min/SBA plate
**Reproducibility**	Lower operator independence (for manual CFU counting) than 96- and 384-wells L-SBA	High operator independence	Higher operator independence than 96-well L-SBA
**Throughput**	Plates/day: 2	Plates/day: 8	Plates/day: 4 (equivalent to sixteen 96-wells plates)
	1 operator/1.5 day: 22 individual sera in single	1 operator/day: 88 individual sera in single	1 operator/day: 188 individual sera in single
**Reagent Costs**	4 EUR/serum	12 EUR/serum	8 EUR/serum

## Data Availability

Data is contained within the article.
